# Production of Cellobionate from Cellulose Using an Engineered *Neurospora crassa* Strain with Laccase and Redox Mediator Addition

**DOI:** 10.1371/journal.pone.0123006

**Published:** 2015-04-07

**Authors:** Amanda Hildebrand, Takao Kasuga, Zhiliang Fan

**Affiliations:** 1 Department of Biological and Agricultural Engineering, University of California, Davis, One Shields Avenue, Davis, CA, 95616, United States of America; 2 Department of Plant Pathology, University of California, Davis, One Shields Avenue, Davis, CA, 95616, United States of America; 3 United States Department of Agriculture—Agricultural Research Service, Davis, CA, 95616, United States of America; University of Huddersfield, UNITED KINGDOM

## Abstract

We report a novel production process for cellobionic acid from cellulose using an engineered fungal strain with the exogenous addition of laccase and a redox mediator. A previously engineered strain of *Neurospora crassa* (F5∆*ace-1*∆*cre-1*∆*ndvB*) was shown to produce cellobionate directly from cellulose without the addition of exogenous cellulases. Specifically, *N*. *crassa* produces cellulases, which hydrolyze cellulose to cellobiose, and cellobiose dehydrogenase (CDH), which oxidizes cellobiose to cellobionate. However, the conversion of cellobiose to cellobionate is limited by the slow re-oxidation of CDH by molecular oxygen. By adding low concentrations of laccase and a redox mediator to the fermentation, CDH can be efficiently oxidized by the redox mediator, with in-situ re-oxidation of the redox mediator by laccase. The conversion of cellulose to cellobionate was optimized by evaluating pH, buffer, and laccase and redox mediator addition time on the yield of cellobionate. Mass and material balances were performed, and the use of the native *N*. *crassa* laccase in such a conversion system was evaluated against the exogenous *Pleurotus ostreatus* laccase. This paper describes a working concept of cellobionate production from cellulose using the CDH-ATBS-laccase system in a fermentation system.

## Introduction

The development of microbial fermentation platforms for the production of organic acids has gained interest in the last decade [1,2] due to the reliability and cost-effectiveness of such processes [3]. In recent years, carboxylic acids, such as lactobionic acid (LBA), have emerged as specialty acids due to their unique physiochemical properties. LBA is a high value-added organic acid, with numerous applications that span the pharmaceutical, food, and cosmetics industries [4]. In order to compete with petroleum-based processes for the production of carboxylic acids, the development of microbial processes utilizing low-cost substrates is essential [4]. LBA is currently produced through chemical synthesis in an energy-intensive process requiring costly metal catalysts. Alternatively, LBA can be produced biologically by various bacterial and fungal strains using refined lactose as the substrate [5–8]. The inexpensive substrate cheese whey was also investigated as a substrate for LBA production by *Psuedomonas taetrolens* in an environmentally-friendly fermentation process [4,7,8]. The key enzyme which catalyzes the biotransformation is lactose dehydrogenase [5–8].

LBA could also be produced from lactose enzymatically from CDH. CDH is a hemoflavoenzyme produced by several cellulolytic fungi. It contains a C-terminalflavin adenine dinucleotide (FAD) domain responsible for oxidizing lactose or cellobiose, resulting in the formation of lactobionate or cellobionate, respectively. The two electrons are subsequently transferred from the FAD domain to the N-terminal heme domain [9]. In order for CDH to regain functionality, the reduced heme domain must be oxidized with the help of an electron acceptor. Oxygen is the electron acceptor in this system if no other electron acceptors are present. Although the overall reaction is thermodynamically favorable, the rate of re-oxidation of CDH by molecular oxygen is very slow and is the rate-limiting step in converting lactose to LBA [10,11].

Other than oxygen, a wide variety of substrates such as metal ions, quinones, and organic dyes can be alternative electron acceptors for the heme domain of the CDH [12]. Dichlorophenolindophenol (DCPIP) and 2,2’-azino-bis[3-ethylbenzothiazoline-6-sulphonic acid] (ABTS) are two redox mediators that can accept electrons from CDH very efficiently [12]. However, the addition of redox mediators to facilitate electron transfer in CDH to improve the conversion rate of lactose to LBA is cost prohibitive unless the redox mediator can be regenerated in-situ.

**Fig 1:**
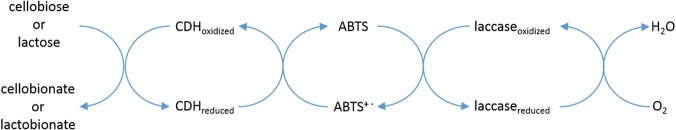
Enzymatic oxidation of substrate (cellobiose/lactose) by CDH. The reduced CDH is oxidized by a redox mediator (ABTS), which is in turn oxidized by laccase. The reduced laccase is oxidized by oxygen, with water as the only byproduct.

Baminger et al. reported a novel CDH-ABTS-laccase bi-enzyme system for fast oxidation of lactose to LBA [12]. Laccases are important multicopper oxidases which are also widely distributed in wood degrading fungi [13]. They are especially prevalent in white rot and brown rot fungi, with speculative involvement in lignin degradation [14]. In contrast to CDH, laccases oxidize a large number of reduced substances and use oxygen as the final electron acceptor very efficiently. One strategy to increase the rate of lactose oxidation by CDH with oxygen as the final electron acceptor is to employ catalytic amounts of DCPIP or ABTS with in-situ regeneration of the redox mediator by laccase. As shown in Fig 1, CDH is reduced. When lactose is oxidized to lactobionic acid, in turn, the reduced CDH is re-oxidized with the help of a redox mediator, which is then regenerated through oxidation by laccase. Lastly, laccase is regenerated when the electrons are passed to oxygen, the final electron acceptor. Such a bi-enzyme cascade system was found to be able to drastically boost the rate of conversion of lactose to LBA using ABTS as a redox mediator [12,15].

Cellulosic biomass, which is available at low cost and in widespread abundance [16], is a potential alternative substrate for the bio-production of carboxylic acids. In this study, we investigate a novel process for the production of cellobionic acid (CBA), which is a sister carboxylic acid (stereoisomer) to LBA, directly from cellulose using an engineered *Neurospora crassa* strain with exogenous laccase and redox mediator addition.

In our previous study, *N*. *crassa* was engineered to produce cellobiose from cellulose by deleting six out of seven β-glucosidase (BGL) genes, resulting in a strain designated F5 [17,18]. The strain also produces CDH, which can oxidize cellobiose to CBA. The strain was further engineered to prevent CBA consumption by knocking out the cellobionate phosphorylase (*ndvB*) gene, and cellulase expression was improved by deleting carbon catabolite repression genes, *cre-1* and *ace-1*, in a strain designated F5Δ*ace-1*Δ*cre-1*Δ*ndvB* [19]. From 20 g/L Avicel, the F5Δ*ace-1*Δ*cre-1*Δ*ndvB* strain produces 20 mM (7 g/L) cellobiose and 10 mM CBA (3.5 g/L). In such a system, oxygen was the final electron acceptor. The re-oxidization of CDH by oxygen is the rate limiting step, which led to incomplete conversion of most of the cellobiose to CBA.

In this study, we explore the possibility of using the engineered *N*. *crassa* F5Δ*ace-1*Δ*cre-1*Δ*ndvB* strain to break down cellulose to cellobiose and produce CDH. Exogenous addition of laccase and ABTS will complement CDH to form the bi-enzyme cascade system to convert cellobiose to CBA. Fermentation conditions including, pH, buffer, and laccase and ABTS concentration and addition times were optimized to maximize the yield of CBA from cellulose. In addition, a material balance on the overall fermentation is included. Lastly, the possibility of using the native *N*. *crassa* laccase in the CDH-ABTS-laccase system was investigated.

## Materials and Methods

### Strains and reagents

Wild type *Neurospora crassa* (2489) was obtained from the Fungal Genetics Stock Center (FGSC) [20]. The F5 strain used in this study is strain 2489 with six out of seven of its *bgl* genesknocked out [17,18]. The F5 strain was engineered for increased cellulase expression and cellulose hydrolysis by knocking out the *ace-1*, *cre-1*, and *ndvB* genes as described previously [19]. The strains used in this study and their sources are listed in Table 1.

**Table 1:** Strains used in this study.

Strain	Genotype	Source
FGSC 2489	Wild type	Fungal genetic source center
F5Δace-1Δcre-1ΔndvB	*bgl-1*::*hph bgl-2*::*hph bgl-3*::*hph bgl-4*::*hph bgl-6*::*hph bgl-7*::*hph mus-51*::*ace-1*::*six cre-1*::*six mat A*	[18]

Laccase from *P*. *ostreatus* was obtained from Sigma Aldrich and used in any studies requiring the exogenous addition of laccase. ABTS was obtained from Sigma Aldrich. CDH used in the study was a recombinant *N*. *crassa* CDH produced by an engineered *Pichia pastoris* strain. It was produced according to the method described by Zhang et al. [21].

### Conversion of cellobiose to CBA using the CDH-ABTS-laccase system

To test the suitability of the CDH-laccase-ABTS redox system on converting cellobiose to cellobionate, a concentration of approximately 30 mM cellobiose was added to 50 mL falcon tubes containing laccase, ABTS, CDH, and buffer at the indicated concentrations and pH. When investigating the effect of pH, sodium citrate buffer at a concentration of 50 mM was used for acidic conditions, sodium phosphate buffer was used at a concentration of 50 mM for basic conditions, and 1x Vogel’s salts medium with no pH adjustment was used for the pH 6 condition.

### Fermentation experiments

*N*. *crassa* strains were grown on agar with 1x Vogel’s salts and 1.5% sucrose in an incubator at 30°C with light. After 3 days, flasks were removed from the incubator and grown for 7 days at room temperature. After a total of 10 days of growth, the conidia were harvested in DI water and filtered through eight layers of cheese cloth. Fermentation experiments were conducted in 250 mL unbaffled flasks with a 50 mL working volume, 1x Vogel’s salts medium, 0.5 g/L of glucose to initiate growth, and 20 g/L Avicel. Conidia were inoculated at a volume to yield a final OD_420_ of 0.15. Flasks were incubated at 28°C in a rotary shaker at 200 rpm with light. Exogenous laccase and ABTS were added to the flasks as indicated in the text. Samples were taken at various time intervals to measure cellobiose or cellobionate concentration. To investigate the effect of starting pH on cellobionate yield, sodium citrate, sodium phosphate, potassium phosphate, and sodium-potassium phosphate buffers of different concentrations were added to achieve the pH indicted. To study the effect of laccase and ABTS addition time on cellobionate production, laccase and ABTS were added to the fermentation broth at final concentrations of 0.05 U/mL and 0.01 mM.

### Laccase production by *Neurospora crassa*

The wild type strain was grown in Vogel’s salts medium on 1.5% sucrose. Cycloheximide, an inducer for laccase production, was added at 48 hours at a final concentration of 3 μM according to literature [22–24]. Laccase expression was monitored according to the assay described below in the “enzyme concentration” section. After an additional 142 hours of fermentation, the broth was filtered to remove residual Avicel and cells in order to obtain the native laccase for evaluation in the CDH-ABTS-laccase redox system in cell free experiments.

### Sample analysis

Concentrations of cellobiose and cellobionate in the cell free experiments and in the fermentation broth were analyzed using a Shimadzu HPLC equipped with a CARBOSep COREGEL-87C (Transgenomic, San Jose, CA, USA) column. Four millimolar calcium chloride at a flow rate of 0.6 mL/min was used as the mobile phase.

### Enzyme concentration

Laccase activity was measured by monitoring the increase in absorbance of ABTS as described previously with minor modifications [15,25]. The reaction mixture contained 5 mM ABTS in 100 mM sodium acetate buffer, pH 4.5. One unit of laccase activity is defined as the amount of enzyme oxidizing 1 μmol of ABTS per minute under the above reaction conditions.

The concentration of CDH was determined by monitoring the decrease in absorbance of DCPIP at 520 nm in a spectrophotometer according to previously established methods with slight modification [15,25]. The reaction contained 0.1 mM DCPIP, 3 mM cellobiose, and 4 mM sodium fluoride in 100 mM sodium acetate buffer at pH 4.5. One unit of enzyme activity is defined as the amount of enzyme reducing 1 μmol of DCIP per minute under the above reaction conditions.

### Mycelial biomass measurements

The dry weight of the of mycelia contained in the fermentation samples was measured by extracting ergosterol from the mycelia and measuring the amount by HPLC [26]. The fermentation residues were collected by filtration through a 0.8 μm membrane. All the residue including the mycelia were harvested, frozen in liquid nitrogen for 1 hour, and ethanol (6 mL) was added to the frozen sample and incubated at 37°C for 2 hours with shaking. An aliquot of KOH solution (60% w/v, 0.8 mL) was added to the mixture, which was then heated to 97°C for 20 min. This sample was cooled and neutralized with HCl (36.5%, ~0.7 mL). The solution was extracted with hexane (3 x 5 mL), the hexane fractions were combined, and air was used to evaporate the solvent. The residue was dissolved in ethanol (1 mL), filtered through a 0.22 μm membrane filter and analyzed by HPLC with PDA detector on a reverse phase column (ZORBAX Eclipse Plus C18, 4.6 x 250 mm, 5 μm particle size, Agilent) and eluted at 1.0 mL/min with methanol-water (97:3 v/v). The amount of biomass was quantified using a standard curve prepared with known *N*. *crassa* dry biomass. The amount of residual Avicel was calculated by subtracting the mycelial biomass from the dry weight of the fermentation residues.

## Results and Discussion

### pH requirement for conversion of cellobiose to CBA using the CDH-ABTS-laccase system

**Fig 2:**
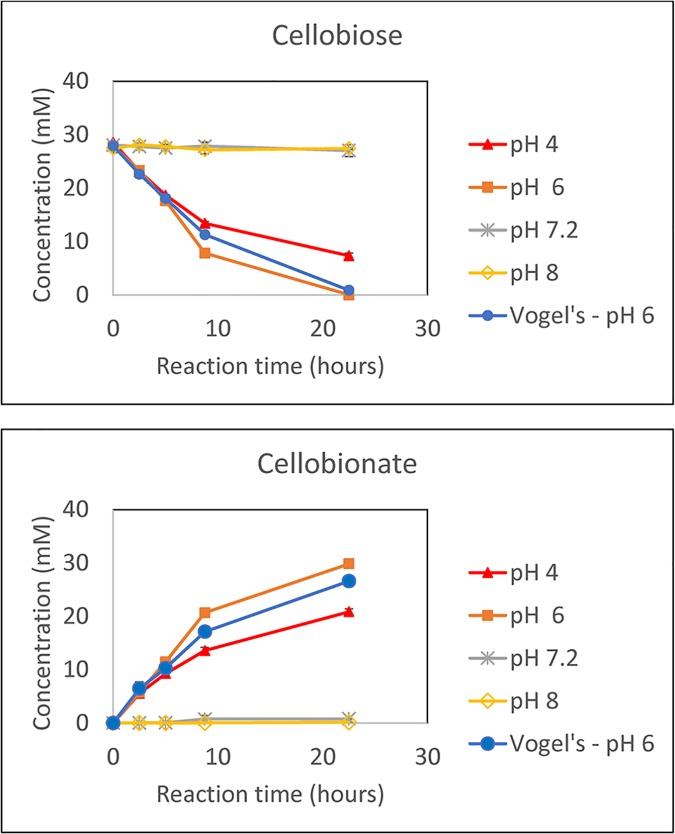
pH effect on the conversion of cellobiose to CBA via CDH-ABTS-laccase mediated conversion. Acidic conditions (pH 4 and pH 6) used 50 mM sodium citrate buffer; pH 7.2 and pH 8 used 50 mM sodium phosphate buffer. The Vogels pH 6 condition used 1x Vogel’s salts medium. The results shown are the means of biological duplicates with the error bars representing the standard deviation.

Acidic, neutral, and basic conditions were tested to determine the effect of pH on the conversion of cellobiose to CBA. The time course of the conversion of cellobiose to CBA is shown in Fig 2. For conditions at pH 6, conversion was completed within 24 hours with an average of 27.9 mM cellobiose converted to 28.6 mM CBA, resulting in an approximate 1:1 molar conversion, as expected. The data obtained support the efficacy of CDH-ABTS-laccase system for converting cellobiose to cellobionate. However, it strictly requires an acidic conditions for the efficient conversion of cellobiose to CBA with the specific CDH and laccase used in this study.

### The effect of starting pH on CBA production

**Fig 3:**
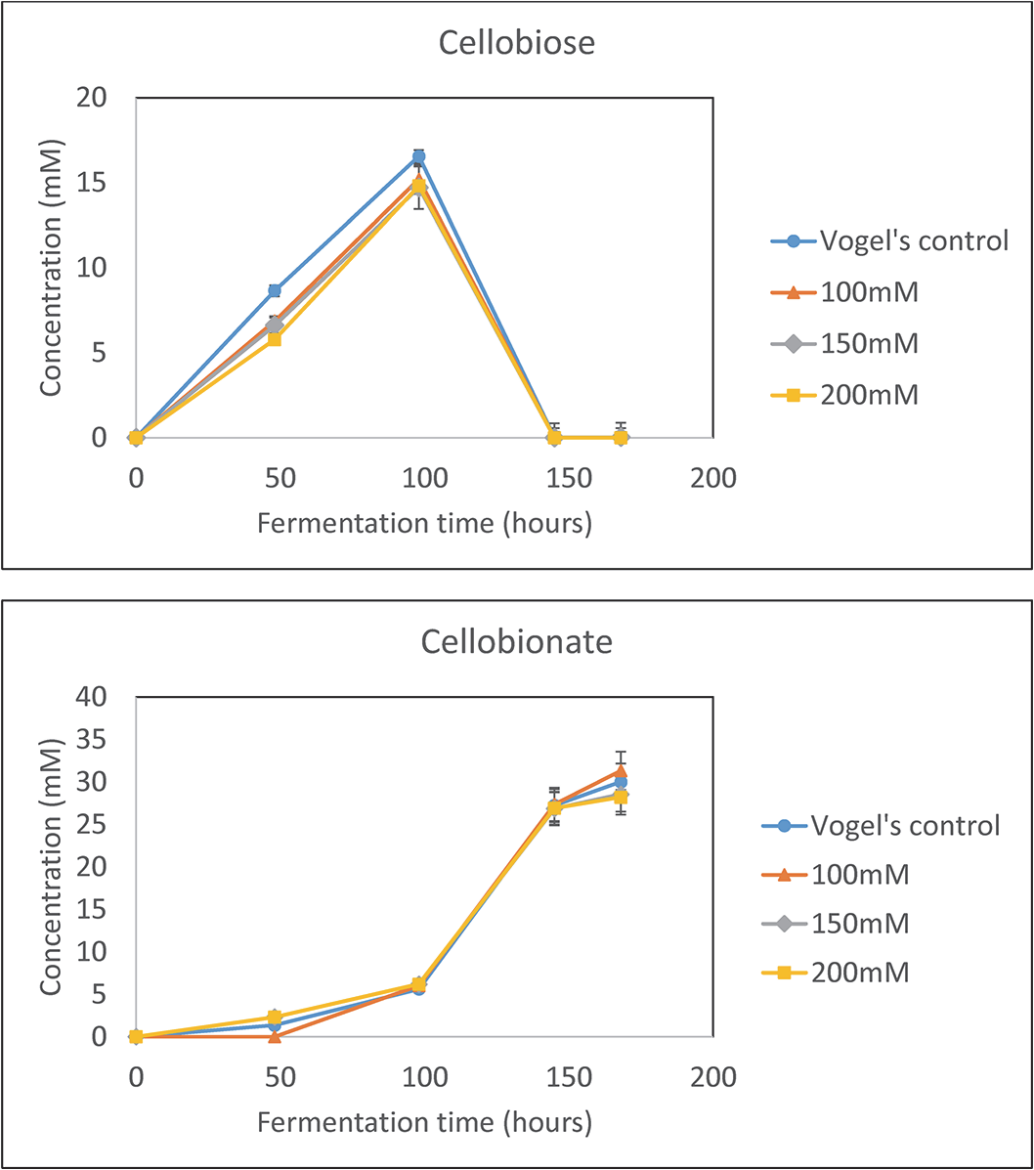
The effect of potassium phosphate buffer concentration and the conversion of cellobiose to CBA via CDH-ABTS-laccase mediated conversion with the *F5Δace-1Δcre-1ΔndvB* strain in 1x Vogel’s medium and 20 g/L Avicel. The values shown are the means of biological duplicates with the error bars representing the standard deviations.

When *F5*Δ*ace-1*Δ*cre-1*Δ*ndvB* is grown on 20 g/L Avicel, approximately 20 mM of cellobiose is produced along with 10 mM CBA. If laccase and ABTS are added to convert cellobiose to CBA, 30 mM of CBA is produced [19]. Because of the high level of CBA production, the pH rapidly drops from pH 6 to pH 4. Buffering the fermentation medium was evaluated to determine the effect of this pH drop on the conversion of cellobiose to CBA. Three different concentrations of potassium phosphate buffer were evaluated to control the rate of pH drop and compared to the unbuffered Vogel’s medium (Fig 3). In the case of the unbuffered Vogel’s medium, the pH drops to 4 over the course of the fermentation. The addition of sodium phosphate buffer results in a slower decrease in pH and higher final pH as the buffer concentration increases. 200 mM potassium phosphate held the pH at 6 until the last day of fermentation where it dropped to 5.5. Although pH varied across the conditions tested, cellobiose and CBA production were not significantly affected. As a result, unbuffered Vogel’s medium was used for the remainder of the fermentation experiments in this study.

### Production of the CBA with laccase and ABTS addition at different times

**Fig 4:**
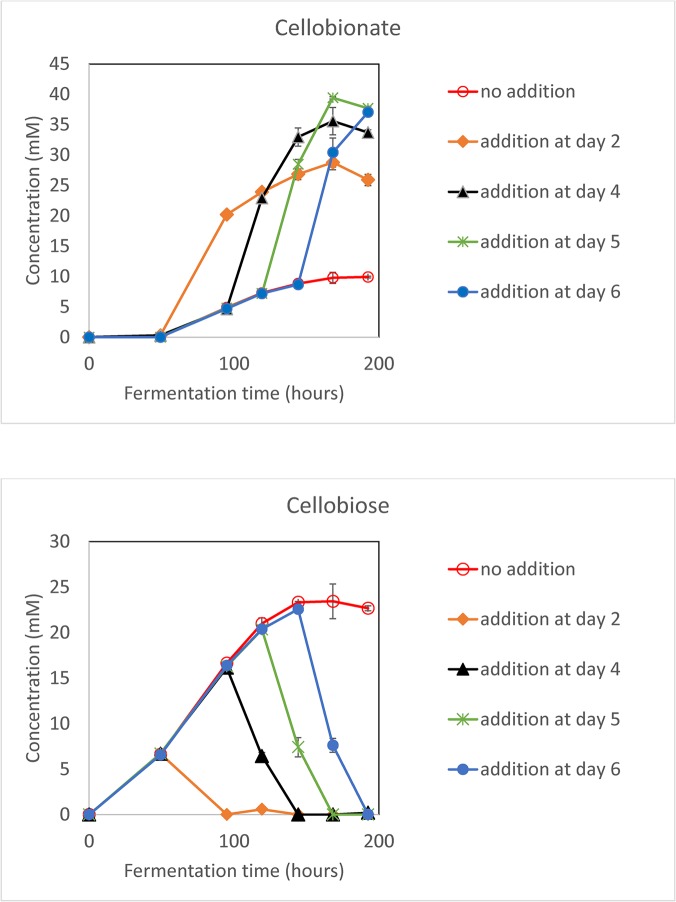
Optimization of laccase (0.05 U/mL) and ABTS (0.01 mM) addition time with the *F5Δace-1Δcre-1ΔndvB* strain grown in 1x Vogel’s medium and 20 g/L Avicel. The values shown are the means of biological triplicates with the error bars representing the standard deviations.

The addition of laccase and ABTS to the fermentation system employing the *F5*Δ*ace-1*Δ*cre-1*Δ*ndvB* strain on 20g/L Avicel was optimized. 0.05 U/mL of laccase and 0.01 mM ABTS were added at various time points. As shown in Fig 4, the cellobiose is completely converted to CBA within 48 hours for all addition times, with an optimal addition time at 120 hours into the fermentation. The maximum CBA concentration occurs at 168 hours with a slight decrease after that for all cases. When no laccase and ABTS are added, maximum cellobiose concentration also occurs 168 hours into the fermentation, and cellobionate production reaches a plateau at that time point as well.

In a parallel experiment with laccase and ABTS addition 120 hours into the fermentation, flasks were harvested at 168 hours to quantify biomass production and Avicel utilization. The results, shown in Table 2, indicate that 67% of the Avicel is hydrolyzed, with 91% going toward CBA production and 4% going toward cell mass production. In the control case, where no laccase and ABTS are added, 62% of the Avicel is hydrolyzed, and a smaller fraction of the consumed Avicel goes toward CBA and cell mass production (29% and 3.3%, respectively).

The higher cellulose conversion in the case of with laccase and ABTS addition indicates that the conversion of cellobiose to CBA may relieve some cellulase inhibition by cellobiose, allowing for hydrolysis of the cello-oligosaccharides and subsequent conversion to CBA.

**Table 2:** Percentage of Avicel hydrolyzed and the percentage directed toward fermentable products for the F5Δ*ace-1*Δ*cre-1*Δ*ndvB* strain grown on 20 g/L Avicel.

	StartingAvicel(g)	ResidualAvicel(g)	CelluloseConversion(%)	Myceliumproduced(g)	Yield ofcellobionate fromconsumed Avicel(mol/mol×100%)	Yield ofmyceliummass fromconsumedAvicel(g/g×100%)
F5Δ*ace-1*Δ*cre-1*Δ*ndvB*With laccase/ABTS	1.00	0.33±0.008	67±0.8%	0.03±0.001	91±4%	4.0±0.2%
F5Δ*ace-1*Δ*cre-1*Δ*ndvB*No laccase/ABTS	1.00	0.38±0.002	62±0.2%	0.02±0.001	29±1%	3.3±0.2%

Errors are calculated based upon standard deviations and error propagation theory.

### Native laccase from *N*. *crassa*

In experiments where exogenous laccase is added to the fermentation, the laccase host was *Pleureotus ostreatus*, a basidiomycete that expresses high levels of laccase [27,28]. While *N*. *crassa* does have a native laccase, it is not naturally expressed except under stress conditions not suitable for efficient fermentation. We investigated the suitability of using the native laccase in the CDH-ABTS-laccase system for conversion of cellobiose to CBA.

**Fig 5:**
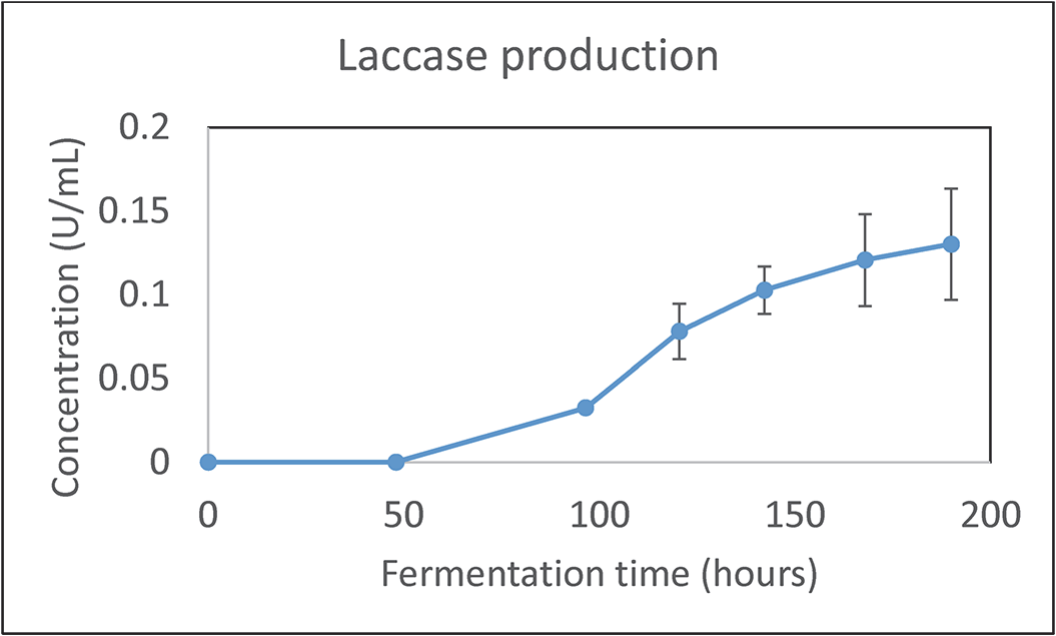
Laccase production by *N*. *crassa* with induction by 3 μM cycloheximide induction at 48 hours into the fermentation. Results shown are the means of biological triplicates with the error bars representing the standard deviations.

Cycloheximide, D-phenylalanine, and copper sulfate are possible inducers for the *N*. *crassa* laccase according to previous studies [22–24,29]. To induce laccase in the *N*. *crassa* F5 strain, 3 uM cycloheximide was added after 48 hours of fermentation. After an additional 142 hours of fermentation, 0.15 U/mL of laccase was obtained, a suitable concentration to test in the cellobiose conversion system as shown in Fig 5.

**Fig 6:**
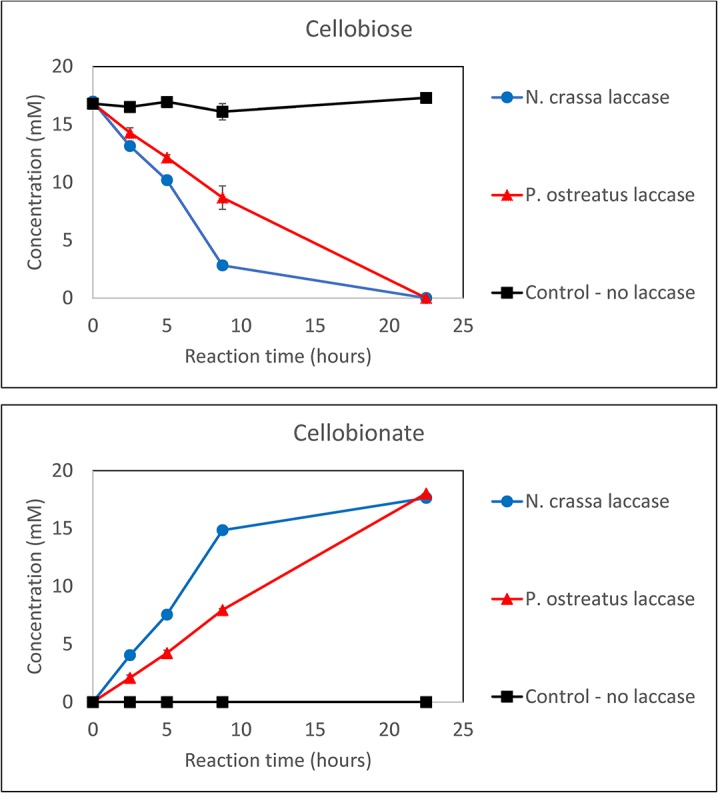
Comparison of the *N*. *crassa* laccase to the *P*. *ostreatus* laccase in the conversion of cellobiose to CBA using the CDH-ABTS-laccase conversion system. The data shown are the means of biological duplicates with the error bars representing the standard deviations.

The produced laccase was tested against the *P*. *ostreatus* laccase in a falcon tube experiment (no cells), where cellobiose, CDH, and ABTS were added and the conversion of cellobiose to CBA monitored as shown in Fig 6. The results indicate that the two laccases have comparable activities, both allowing for efficient conversion of cellobiose to CBA in the CDH-ABTS-laccase conversion system.

Cellulolytic fungi such as *N*. *crassa* can potentially produce all the enzymes needed to convert cellulose to CBA. *N*. *crassa* was able to produce cellulases and CDH. With the help of the exogenously added laccase and ABTS, *N*. *crassa* was able to convert cellulose to CBA at very high yield. Our preliminary study showed that the native laccase produced by *N*. *crassa* works as efficiently as the *P*. *ostreatus* laccase. This opens up the possibility to engineer *N*. *crassa* to produce all the enzymes needed to convert cellulose to CBA. Homologous or heterologous expression of laccase in *N*. *crassa* could be achieved by engineering the native or heterologous laccase for expression under a constitutive or inducible promoter, allowing it to be produced under the tested fermentation conditions.

Literature has reported over-expressing the native in *N*. *crassa* at adequate concentrations for efficient conversion of cellobiose to CBA in the presence of CDH and ABTS (55 mg/L) [30]. Repeating these results in *F5*Δ*ace-1*Δ*cre-1*Δ*ndvB* is a possibility. Alternatively, one study showed that a laccase de-repressed mutant *lah-1* produced laccase at levels even higher than when the wild type was induced with cycloheximide [31]. The de-repression was a result of a single mutation in an unknown gene mapped between *nit-2* and *leu-3* in linkage group I. Similarly de-repressing the *F5*Δ*ace-1*Δ*cre-1*Δ*ndvB* strain or overexpressing laccase in this strain could create a strain which would require only the addition of a low concentration of ABTS to produce CBA from cellulose and will be a focus of our ongoing work in this area.

ABTS was used as the redox mediator in the tested system. Although it is only needed in catalytic amounts, it is very expensive for industrial applications and an alternative low cost redox mediator must be used. A wide variety of inorganic metals and organic dyes can be the alternative redox mediator [32]. Lignin degradation products such as vanillin, ferulic acid or *p*-coumaric acid, which are generated as waste in the paper and pulp industry, have been demonstrated to be very efficient naturally-occurring laccase mediators [33–36]. They could be cheap alternative redox mediator sources [32]. If the pretreated lignocellulosic biomass was used as the substrate instead of pure cellulose, these compounds could naturally exist in the feedstock stream and the exogenous addition of redox mediator could potentially be avoided.

## Conclusion

CBA can be produced from cellulose by an engineered *N*. *crassa* strain with exogenous laccase addition. The native laccase produced by *N*. *crassa* functioned as well as the exogenously added laccase. The *N*. *crassa* strain has the potential to produce all the enzymes needed for microbial conversion of cellulose to CBA. The conversion concept is applicable to other industrially relevant cellulolytic fungi for CBA production from cheap feedstocks such as cellulosic biomass.
